# Long-Term Symptoms Associated With SARS-CoV-2 Infection Among Blood Donors

**DOI:** 10.1001/jamanetworkopen.2024.5611

**Published:** 2024-04-08

**Authors:** Melisa M. Shah, Bryan R. Spencer, Jade James-Gist, James M. Haynes, Leora R. Feldstein, Susan L. Stramer, Jefferson M. Jones, Sharon H. Saydah

**Affiliations:** 1 Coronavirus and Other Respiratory Viruses Division, National Center for Immunization and Respiratory Diseases, Centers for Disease Control and Prevention, Atlanta, Georgia; 2 American Red Cross, Scientific Affairs, Dedham, Massachusetts; 3 American Red Cross, Scientific Affairs, Rockville, Maryland

## Abstract

**Importance:**

Long-term symptoms, lasting more than 4 consecutive weeks after acute COVID-19 disease, are an important consequence of SARS-CoV-2 infection. Many prior studies have lacked a non–SARS-CoV-2–infected control population to distinguish background prevalence of symptoms from the direct impact of COVID-19 disease.

**Objective:**

To examine the prevalence of long-term physical and mental health symptoms associated with SARS-CoV-2 infection in a large population of blood donors based on self-report and serologic test results.

**Design, Setting, and Participants:**

This cross-sectional study included American Red Cross blood donors (aged ≥18 years) who were surveyed between February 22 and April 21, 2022, about new long-term symptoms arising after March 2020 and their SARS-CoV-2 infection status. All participants underwent at least 1 serologic test for antinucleocapsid antibodies between June 15, 2020, and December 31, 2021.

**Exposures:**

SARS-CoV-2 infection as defined by a self-reported, confirmed acute infection or antinucleocapsid antibody positivity.

**Main Outcomes and Measures:**

New long-term symptoms since March 2020, including 5 symptom categories (neurologic, gastrointestinal, respiratory and cardiac, mental health, and other).

**Results:**

Among 818 361 individuals who received the survey, 272 965 (33.4%) responded, with 238 828 meeting the inclusion criteria (138 576 [58.0%] female; median [IQR] age, 59.0 [47.0-67.0] years). Of the 83 015 individuals with a history of SARS-CoV-2 infection, 43.3% reported new long-term symptoms compared with 22.1% of those without a history of SARS-CoV-2 infection. After controlling for age, sex, race and ethnicity, and number of underlying conditions, those with a history of SARS-CoV-2 infection had an increased odds of new long-term symptoms compared with those without (adjusted odds ratio [AOR], 2.55; 95% CI, 2.51-2.61). Female sex and a history of chronic conditions were associated with new long-term symptoms. Long-term symptoms in the other category (AOR, 4.14; 95% CI, 4.03-4.25), which included changes in taste or smell, and the respiratory and cardiac symptom categories (AOR, 3.21; 95% CI, 3.12-3.31) were most associated with prior SARS-CoV-2 infection. Mental health long-term symptoms were also associated with prior SARS-CoV-2 infection (AOR, 1.05; 95%, CI, 1.02-1.08).

**Conclusions and Relevance:**

This study’s findings suggest that long-term symptoms lasting more than 4 weeks are common in the adult population, but there is a significantly higher prevalence among those with SARS-CoV-2 infection. Continued efforts to define and track long-term sequelae of SARS-CoV-2 using a control group without infection and serologic information to include those who had asymptomatic or unidentified infections are needed.

## Key Points

**Question:**

Are US blood donors with a history of SARS-CoV-2 infection (defined by self-report or serologic testing) more likely to have long-term symptoms compared with those without a history of SARS-CoV-2 infection?

**Findings:**

In this cross-sectional study of 238 828 survey participants, 43.3% of those with a history of SARS-CoV-2 infection reported new long-term symptoms compared with 22.1% without a history of infection.

**Meaning:**

These findings suggest that long-term symptoms are common in the adult population, but there is a significantly higher prevalence among those with SARS-CoV-2 infection.

## Teaser

This cross-sectional study examines the presence of long-term physical and mental health symptoms in a large population of blood donors with and without a history of SARS-CoV-2 infection.

## Introduction

Long-term symptoms, lasting more than 4 weeks after acute COVID-19 disease, are an important consequence of SARS-CoV-2 infection. These symptoms, referred to as post–COVID-19 conditions, can significantly reduce quality of life but may not be systematically diagnosed or documented. Prevalence estimates for post–COVID-19 conditions vary widely across studies depending on the definition and method of assessment.^1,2,3,4,5,6^ A meta-analysis reported the pooled global prevalence of post–COVID-19 conditions to be 43% among those with a confirmed COVID-19 diagnosis.^6^ However, prior studies have often lacked a non–SARS-CoV-2–infected control population to distinguish background prevalence of symptoms from the effect of COVID-19 disease. A more robust understanding is needed of how long-term symptoms after SARS-CoV-2 infection differ from baseline levels and how to distinguish them from the physical and mental impacts of the pandemic.

Serologic testing to confirm prior infection may be particularly helpful for the evaluation of post–COVID-19 conditions because many SARS-CoV-2 infections are not detected and many individuals who are asymptomatic or experience mild symptoms may not be tested during the acute phase of infection.^7^ Additionally, persistent symptoms after self-reported COVID-19 are not always associated with serologic evidence of prior infection.^8^ Hence, a high-sensitivity serologic test combined with self-reported infection status maximizes the detection of prior infections and may be advantageous in studies of post–COVID-19 conditions. We examine the presence of long-term physical and mental health symptoms in a large population of blood donors with and without SARS-CoV-2 infection.

## Methods

### Study Design

This survey received approval from the American Red Cross Institutional Review Board. Consent for research is integrated into consent for blood donation. This voluntary survey was approved by the American Red Cross Institutional Review Board under a waiver of consent. It was also reviewed by the Centers for Disease Control and Prevention and conducted consistently with applicable federal law and Centers for Disease Control and Prevention policy. This cross-sectional study followed the Strengthening the Reporting of Observational Studies in Epidemiology (STROBE) reporting guidelines for cross-sectional studies.

### Serologic Testing

The American Red Cross tested all blood donations for SARS-CoV-2 antibodies from June 15, 2020, through June 25, 2021, and a subset of donations from July 5, 2021, through December 31, 2021 (eFigure in Supplement 1). Serologic testing included assays for antinucleocapsid (anti-N) antibodies, which are produced after SARS-CoV-2 infection and not after vaccination. Anti-N antibody positivity was assessed with the Elecsys (Roche Diagnostics) and Vitros (Ortho Clinical Diagnostics) anti-N assays. These antibody tests have high sensitivity to detect infections for at least 1 year after infection.^9,10,11^

### Blood Donation Intake and Electronic Survey

The American Red Cross includes blood donors from 44 states and provides approximately 40% of the US blood supply. At the time of blood donation, data on sex, age, race, and ethnicity were collected. Blood donors self-identify race and ethnicity from fixed categories, including American Indian or Alaska Native, Asian, Black or African American, Hispanic, White, and multiracial or other (any races not listed here). Race and ethnicity data were included to describe the demographics of the population. An electronic survey was then sent (eAppendix in Supplement 1) between February 22 and April 21, 2022, to blood donors who had donated from June 15, 2020, to December 31, 2021, and agreed to email contact. Characteristics of nonresponders were collected and included in eTable 2 in Supplement 1. The survey included questions about prior COVID-19 symptoms and testing, COVID-19 vaccination history (including number of doses), and chronic health conditions. Chronic health conditions present before March 2020 included in this survey were chronic pain, anxiety, depression, chronic headaches or migraines, stroke, kidney disease, liver disease, heart disease or other cardiovascular disease, lung disease or asthma, high blood pressure, diabetes, any immune system disorder, and cancer. The survey also collected information on long-term symptoms since March 2020 that lasted longer than 4 weeks that the participant did not previously have, duration of long-term symptoms, health-seeking behavior, and self-reported general, physical, and mental health (eTable 1 in Supplement 1). For the primary analysis, SARS-CoV-2 infection was defined as a self-reported positive diagnostic test result (swab or saliva) or health care professional diagnosis (surveys administered February 22 through April 21, 2022) or a positive anti-N antibody test result from American Red Cross testing from June 15, 2020, to December 31, 2021. The timing of long-term symptoms in relation to prior SARS-CoV-2 infection was not collected. Long-term symptoms were grouped by system (neurologic, gastrointestinal, respiratory or cardiac, mental health, and other), and the order of questions was randomized to limit bias. The 18- to 34-year age group, unvaccinated blood donors, and those with anti-N antibody positivity comprised a higher proportion of nonresponders compared with responders (eTable 2 in Supplement 1).

### Statistical Analysis

Demographic information, COVID-19 vaccination status, long-term symptoms, and other factors were described using summary statistics. Participants who responded to none of the long-term symptom questions were excluded from the analysis. Continuous variables were expressed as means (SDs), and categorical variables were expressed as numbers (percentages). For univariate analysis, a 2-sided Wilcoxon rank sum test was used for continuous variables, and a 2-sided Pearson χ^2^ test was used for categorical variables. For all analyses, we used *P* ≤ .05 to define statistical significance. The duration of long-term symptoms was determined in a subgroup of participants reporting resolved symptoms at the time of the survey. Adjusted odds ratios (AORs) were calculated to examine the association between long-term symptoms and previous infection, using logistic regression modeling with a random intercept for state of residence. The models were adjusted for sex, age category, race and ethnicity, and number of underlying medical conditions. Age was categorized as 18 to 34, 34 to 44, 45 to 54, 55 to 64, 65 to 74, and 75 years or older. Multiple imputation was used for missing data in the models. Six regression models were performed: 1 for any new long-term symptoms and 1 each for the long-term symptom categories as outcomes (neurologic, gastrointestinal, respiratory and cardiac, mental health, and other). Analyses were conducted using R statistical software, version 2023.03.1 (R Foundation for Statistical Computing).

## Results

### Blood Donor Survey Participants and Serologic Testing

**Figure 1.**
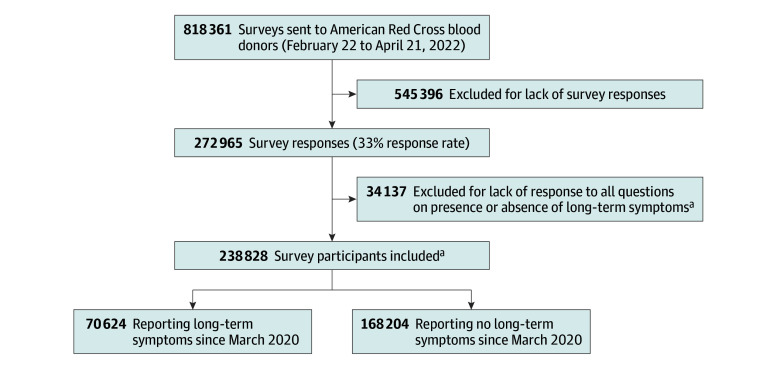
Flowchart of Inclusion Criteria ^a^If participants answered survey questions for at least 1 group of the 5 symptom categories (neurologic, gastrointestinal, respiratory and cardiac, mental health, and other), they were included in the study; 212 318 of 238 828 participants (88.9%) answered questions from all 5 symptom categories.

Among 818 361 adults who received a survey, 272 965 (33.4%) responded (Figure 1; eTable 2 in Supplement 1). Among the participants, 34 137 (12.5%) were excluded for not answering questions on long-term symptoms. Of the remaining 238 828 respondents (median [IQR] age, 59.0 [47.0-67.0 years), 138 576 (58.0%) were female and 100 252 (42.0%) were male, and 586 (0.2%) were American Indian, 4947 (2.1%) Asian, 4333 (1.8%) Black or African American, 5748 (2.4%) Hispanic, 219 984 (92.1%) White, 2780 (1.2%) multiracial or other (Table 1). Among these 238 828 participants, 70 624 (29.6%) reported any new symptoms lasting more than 4 weeks since March 2020 (Figure 1). Compared with those without long-term symptoms, blood donors with long-term symptoms were more likely to be female (47 223 of 70 624 [66.9%] vs 91 353 of 168 204 [54.3%]), younger (median [IQR] age, 55.0 [43.0-64.0] vs 60.0 [50.0-68.0] years), Hispanic (2265 of 70 477 [3.2%] vs 3483 of 167 901 [2.1%]), have 1 or more comorbidity (37 377 of 69 903 [53.5%] vs 70 991 of 166 729 [42.6%]), and report being unvaccinated (8138 of 70 334 [11.6%] vs 14 648 of 167 611 [8.7%]) (Table 1). A greater proportion of those without SARS-CoV-2 infection had received at least 1 vaccination at the time of the survey (147 851 of 153 891 [96.1%] vs 66 111 of 82 538 [80.1%]) (eTable 3 in Supplement 1).

**Table 1.** Demographic Characteristics and History of SARS-CoV-2 Infection Among Blood Donors With and Without at Least 1 Long-Term Symptom (Lasting >4 Weeks) Since March 2020

Characteristic	No./total No. (%) of participants [95% CI]		*P* value^a^
	Reported long-term symptoms (n = 70 624)	Did not report long-term symptoms (n = 168 204)	
Sex			
Female	47 223/70 624 (66.9) [66.5-67.2]	91 353/168 204 (54.3) [54.1-54.5]	<.001
Male	23 401/70 624 (33.1) [32.8-33.5]	76 851/168 204 (45.7) [45.5-45.9]	
Age, median (IQR), y	55.0 (43.0-64.0)	60.0 (50.0-68.0)	<.001
Age category, y			
18-34	8315/70 624 (11.8) [11.5-12.0]	11 610/168 204 (6.9) [6.8-7.0]	<.001
35-44	12 012/70 624 (17.0) [16.7-17.3]	18 660/168 204 (11.1) [10.9-11.2]	
45-54	14 569/70 624 (20.6) [20.3-20.9]	27 397/168 204 (16.3) [16.1-16.5]	
55-64	18 841/70 624 (26.7) [26.4-27.0]	50 120/168 204 (29.8) [29.6-30.0]	
65-74	14 038/70 624 (19.9) [19.6-20.2]	49 853/168 204 (29.6) [29.4-29.9]	
≥75	2849/70 624 (4.0) [3.9-4.2]	10 564/168 204 (6.3) [6.2-6.4]	
Race and ethnicity			
American Indian or Alaska Native	250/70 477 (0.4) [0.3-0.4]	336/167 901 (0.2) [0.2-0.2]	<.001
Asian	1234/70 477 (1.8) [1.7-1.9]	3713/167 901 (2.2) [2.1-2.3]	
Black or African American	1359/70 477 (1.9) [1.8-2.0]	2974/167 901 (1.8) [1.7-1.8]	
Hispanic	2265/70 477 (3.2) [3.1-3.3]	3483/167 901 (2.1) [2.0-2.1]	
White	64 327/70 477 (91.3) [91.1-91.5]	155 657/167 901 (92.7) [92.6-92.8]	
Multiracial and other^b^	1042/70 477 (1.5) [1.4-1.6]	1738/167 901 (1.0) [1.0-1.1]	
Missing	147	303	
≥1 Chronic health conditions^c^	37 377/69 903 (53.5) [53.1-53.8]	70 991/166 729 (42.6) [42.3-42.8]	<.001
Region			
Midwest	24 013/70 569 (34.0) [33.7-34.4]	56 510/168 088 (33.6) [33.4-33.8]	<.001
Northeast	14 358/70 569 (20.3) [20.0-20.6]	37 157/168 088 (22.1) [21.9-22.3]	
South	16 619/70 569 (23.6) [23.2-23.9]	39 993/168 088 (23.8) [23.6-24.0]	
West	15 579/70 569 (22.1) [21.8-22.4]	34 428/168 088 (20.5) [20.3-20.7]	
Missing	55	116	
SARS-CoV-2 vaccination status			
≥1 Vaccine dose	62 196/70 334 (88.4) [88.2-88.7]	152 963/167 611 (91.3) [91.1-91.4]	<.001
Unvaccinated	8138/70 334 (11.6) [11.3-11.8]	14 648/167 611 (8.7) [8.6-8.9]	
Missing	290	593	
History of SARS-CoV-2 by self-report			
Yes, confirmed by diagnostic test or health care practitioner	27 537/70 574 (39.0) [38.7-39.4]	31 469/168 089 (18.7) [18.5-18.9]	<.001
Yes, confirmed only by serologic test	5152/70 574 (7.3) [7.1-7.5]	7425/168 089 (4.4) [4.3-4.5]	
Yes, I think I have been infected but not confirmed by test	3383/70 574 (4.8) [4.6-5.0]	4377/168 089 (2.6) [2.5-2.7]	
No history of infection	31 136/70 574 (44.1) [43.8-44.5]	118 231/168 089 (70.3) [70.1-70.6]	
Unsure if I have been infected	3366/70 574 (4.8) [4.6-4.9]	6587/168 089 (3.9) [3.8-4.0]	
Missing	50	115	
Antinucleocapsid antibody status			
Negative	48 145/70 624 (68.2) [67.8-68.5]	140 571/168 204 (83.6) [83.4-83.7]	<.001
Positive	22 479/70 624 (31.8) [31.5-32.2]	27 633/168 204 (16.4) [16.3-16.6]	
SARS-CoV-2 infection by reported confirmed infection or antinucleocapsid antibody positivity			
Yes	35 927/69 975 (51.3) [51.0-51.7]	47 088/167 323 (28.1) [27.9-28.4]	<.001
No^d^	34 048/69 975 (48.7) [48.3-49.0]	120 235/167 323 (71.9) [71.6-72.1]	
Missing^e^	649	881	

^a^ Pearson χ^2^ test for all except median age, for which Wilcoxon rank sum test was used.

^b^ Indicates any race or ethnicity not otherwise listed.

^c^ Includes chronic pain; anxiety; depression; chronic headaches; stroke; kidney, liver, heart, and lung disease; high blood pressure; diabetes; immune disorder; or cancer.

^d^ Those who were antinucleocapsid antibody negative who reported having been infected but never had a confirmed diagnosis, reported never being infected, or were unsure whether they were ever infected.

^e^ Those who were antinucleocapsid antibody negative without self-reported infection status and those who were antinucleocapsid negative with self-reported infection based on serologic testing.

Among the 238 828 participants, 83 015 (34.8%) had a prior infection, including 50 112 (21.0%) with anti-N antibody positivity and 59 006 (24.7%) with self-reported confirmed infection. Among participants with self-reported SARS-CoV-2 infection, 1106 of 57 783 (2.0%) reported being hospitalized for COVID-19. For those with SARS-CoV-2 infection, 35 927 of 83 015 (43.3%) reported long-term symptoms compared with 34 048 of 154 283 (22.1%) of those without SARS-CoV-2 infection (Table 2). Among 23 979 individuals with no reported prior infection but with anti-N antibodies, 8377 (34.9%) reported long-term symptoms compared with 14 089 of 26 103 (54.0%) with infection confirmed by serologic testing and self-report (eTable 4 in Supplement 1).

**Table 2.** Numbers and Types of Long-Term Symptoms by Serologic and Self-Reported SARS-CoV-2 Status

Symptom	No./total No. (%) of participants		
	Overall (N = 238 828)	Antinucleocapsid antibody positive or reported confirmed infection (n = 83 015)	Antinucleocapsid antibody negative and no reported infection (n = 154 283)^a^
Any persistent symptoms	70 624/238 828 (29.6)	35 927/83 015 (43.3)	34 048/154 283 (22.1)
Median (IQR) No. of symptoms among those with any symptoms	2 (1-3)	2 (1-4)	2 (1-3)
Neurologic symptoms	34 839/231 289 (15.1)	18 949/80 218 (23.6)	15 548/149 586 (10.4)
Fatigue	13 373/231 289 (5.8)	8877/80 218 (11.1)	4357/149 586 (2.9)
Headache	5631/231 289 (2.4)	3389/80 218 (4.2)	2175/149 586 (1.5)
Malaise	2950/231 289 (1.3)	2026/80 218 (2.5)	887/149 586 (0.6)
Difficulty sleeping	12 731/231 289 (5.5)	5531/80 218 (6.9)	7081/149 586 (4.7)
Difficulty speaking	2653/231 289 (1.1)	1714/80 218 (2.1)	909/149 586 (0.6)
Problems with balance	3976/231 289 (1.7)	2109/80 218 (2.6)	1827/149 586 (1.2)
Numbness	6089/231 289 (2.6)	2984/80 218 (3.7)	3033/149 586 (2.0)
Difficulty thinking or concentrating	15 891/231 289 (6.9)	10 150/80 218 (12.7)	5564/149 586 (3.7)
Dizziness	4139/231 289 (1.8)	2432/80 218 (3.0)	1652/149 586 (1.1)
Difficulty swallowing	1098/231 289 (0.5)	581/80 218 (0.7)	507/149 586 (0.3)
Gastrointestinal symptoms	7252/231 164 (3.1)	3664/80 177 (4.6)	3510/149 509 (2.3)
Loss of appetite	3004/231 164 (1.3)	1718/80 177 (2.1)	1249/149 509 (0.8)
Constipation	1989/231 164 (0.9)	902/80 177 (1.1)	1063/149 509 (0.7)
Diarrhea	2193/231 164 (0.9)	1017/80 177 (1.3)	1151/149 509 (0.8)
Vomiting	867/231 164 (0.4)	483/80 177 (0.6)	373/149 509 (0.2)
Abdominal pain	1764/231 164 (0.8)	925/80 177 (1.2)	813/149 509 (0.5)
Respiratory and cardiac symptoms	21 182/231 170 (9.2)	12 639/80 067 (15.8)	8304/149 628 (5.5)
Congestion	8240/231 170 (3.6)	4329/80 067 (5.4)	3820/149 628 (2.6)
Cough	8530/231 170 (3.7)	5301/80 067 (6.6)	3120/149 628 (2.1)
Shortness of breath	6012/231 170 (2.6)	4274/80 067 (5.3)	1653/149 628 (1.1)
Palpitations	4162/231 170 (1.8)	2580/80 067 (3.2)	1534/149 628 (1.0)
Throat pain	1862/231 170 (0.8)	970/80 067 (1.2)	873/149 628 (0.6)
Chest pain	2466/231 170 (1.1)	1639/80 067 (2.0)	794/149 628 (0.5)
Other symptoms	28 399/231 027 (12.3)	18 525/80 244 (23.1)	9559/149 310 (6.4)
Bruising	1105/231 027 (0.5)	655/80 244 (0.8)	431/149 310 (0.3)
Menstruation changes^b^	3651/133 795 (2.7)	1917/48 185 (4.0)	1698/84 852 (2.0)
Chills	879/231 027 (0.4)	501/80 244 (0.6)	372/149 310 (0.2)
Hair loss	6396/231 027 (2.8)	4430/80 244 (5.5)	1889/149 310 (1.3)
Joint swelling	2034/231 027 (0.9)	1172/80 244 (1.5)	844/149 310 (0.6)
Joint pain	8778/231 027 (3.8)	4705/80 244 (5.9)	3982/149 310 (2.7)
Skin changes	2411/231 027 (1.0)	1219/80 244 (1.5)	1162/149 310 (0.8)
Weight loss	1517/231 027 (0.7)	767/80 244 (1.0)	727/149 310 (0.5)
Change in taste	8809/231 027 (3.8)	7739/80 244 (9.6)	955/149 310 (0.6)
Change in smell	9794/231 027 (4.2)	8800/80 244 (11.0)	868/149 310 (0.6)
Mental health symptoms	24 522/232 723 (10.5)	9634/80 639 (11.9)	14 694/150 591 (9.8)
Anxiety	15 278/232 723 (6.6)	6042/80 639 (7.5)	9103/150 591 (6.0)
Depression	10 445/232 723 (4.5)	4220/80 639 (5.2)	6131/150 591 (4.1)
PTSD	1897/232 723 (0.8)	853/80 639 (1.1)	1025/150 591 (0.7)
Mood changes	11 157/232 723 (4.8)	4568/80 639 (5.7)	6502/150 591 (4.3)

Abbreviation: PTSD, posttraumatic stress disorder.

^a^ Excludes 1530 participants, including those who were antinucleocapsid antibody negative and without self-reported infection status and those who were antinucleocapsid negative with self-reported infection based on serologic testing.

^b^ This row of denominators includes only women who reported at least 1 long-term symptom.

The median (IQR) date of first anti-N antibody positivity among those with antibodies was March 11, 2021 (January 7 to May 18, 2021). The median (IQR) date of last serologic testing among those without anti-N antibodies was November 4, 2021 (September 4 to December 6, 2021). Among those reporting a date of prior SARS-CoV-2 infection, the median (IQR) time between infection and the survey response was 334 (79-479) days.

### Long-Term Symptom Categories and Duration

Among donors with prior SARS-CoV-2 infection, 18 949 of 80 218 (23.6%) reported neurologic symptoms, 18 525 of 80 244 (23.1%) reported other symptoms (including changes in taste or smell), 12 639 of 80 067 (15.8%) reported respiratory or cardiac symptoms, 9634 of 80 639 (11.9%) reported mental health symptoms, and 3664 of 80 177 (4.6%) reported gastrointestinal symptoms (Table 2). The 2 most common long-term symptoms overall among those with SARS-CoV-2 infection were difficulty thinking or concentrating (10 150 of 80 218 [12.7%]) and fatigue (8877 of 80 218 [11.1%]). Among those without SARS-CoV-2 infection, anxiety was the most common long-term symptom (9103 of 150 591 [6.0%]). Among those with SARS-CoV-2 infection, those reporting symptoms in the other category were the most likely to attribute their long-term symptoms to COVID-19 (11 407 of 18 119 [ 63.0%]), whereas those reporting mental health symptoms were least likely to do so (3728 of 9320 [ 40.0%]) (eTable 5 in Supplement 1).

**Figure 2.**
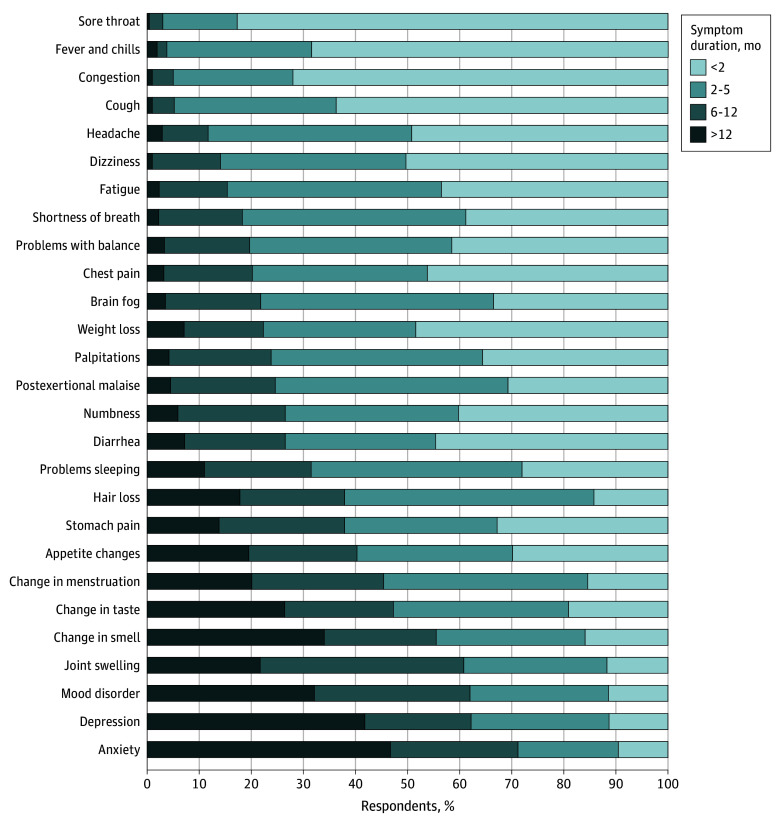
Duration of Specific Symptoms Among Blood Donors With a History of SARS-CoV-2 by Self-Report or Serologic Testing Who Reported Having Any Symptoms Lasting More Than 4 Weeks Since March 2020 That Were Resolved at the Time of the Survey Symptoms are ordered by increasing percentage of participants reporting that the symptom lasted 6 months or longer. Symptoms that occurred in 50 participants or fewer were excluded from this figure due to small sample size. These symptoms included vomiting, constipation, problems speaking, unusual bruising, skin changes, joint or muscle pain, and posttraumatic stress disorder.

There were 13 799 resolved symptoms for which a duration was reported among those with a history of SARS-CoV-2 infection; the proportion of mental health symptoms (anxiety, depression, and mood disorders) lasting longer than 6 months was higher than that of other symptoms (Figure 2). Among SARS-CoV-2–infected participants with resolved fatigue, a prominent long COVID-19 symptom,^12^ 39 of 1682 (2.3%) reported fatigue lasting longer than 12 months, 220 of 1682 (13.1%) reported fatigue lasting 6 to 12 months, 692 of 1682 (41.1%) reported fatigue lasting 2 to 5 months, and 731 of 1682 (43.5%) reported fatigue lasting less than 2 months. Most participants reporting sore throat, fever and chills, congestion, and cough reported that their symptoms lasted fewer than 2 months. Among those with ongoing symptoms at the time of the survey, difficulty thinking and concentrating, fatigue, and cough were the most common symptoms (eTable 6 in Supplement 1).

### Factors Associated With Long-Term Symptoms

After controlling for sex, age, race and ethnicity, and number of underlying chronic conditions before March 2020, SARS-CoV-2 infection was associated with 2.55 (95% CI, 2.51-2.61) increased odds of reporting at least 1 long-term symptom compared with those without SARS-CoV-2 infection (Table 3). Long-term symptoms in the other category (AOR, 4.14; 95% CI, 4.03-4.25), which included changes in taste or smell, and the respiratory and cardiac symptom category (AOR, 3.21; 95% CI, 3.12-3.31) were most associated with prior SARS-CoV-2 infection. Long-term mental health symptoms were also associated with prior SARS-CoV-2 infection (AOR, 1.05; 95% CI, 1.02-1.08). Female sex (AOR, 1.55; 95% CI, 1.52-1.58) was associated with any long-term symptoms as well as individual symptoms across the 5 symptom groups. Increasing number of chronic conditions was associated with any new long-term symptoms (≥4 chronic conditions; AOR, 4.03; 95%, CI 3.79-4.28). Age categories had heterogeneous associations across the 5 symptom groups. Age was not associated with the presence of new respiratory or cardiac symptoms but was strongly associated with new long-term mental health symptoms (AOR, 0.16; 95% CI, 0.14-0.17 for age ≥75 years compared with 18-34 years). Patients of Hispanic ethnicity (AOR, 1.21; 95% CI, 1.14-1.28), American Indian race (AOR, 1.46; 95% CI, 1.23-1.73), or other race and ethnicity (AOR, 1.22; 95% CI, 1.12-1.32) had an increased odds of any long-term symptoms compared with White participants.

**Table 3.** Factors Associated With Any Long-Term Symptoms and Specific Symptom Categories, American Red Cross Blood Donors, February to April 2022^a^^,^^b^

Factor	AOR (95% CI)					
	Any new long-term symptom	Neurologic symptoms	Gastrointestinal symptoms	Respiratory and cardiac symptoms	Other symptoms	Mental health symptoms
Prior confirmed SARS-CoV-2 infection^c^						
Yes	2.55 (2.51-2.61)	2.60 (2.54-2.66)	1.91 (1.82-2.01)	3.21 (3.12-3.31)	4.14 (4.03-4.25)	1.05 (1.02-1.08)
No	1.0 [Reference]	1.0 [Reference]	1.0 [Reference]	1.0 [Reference]	1.0 [Reference]	1.0 [Reference]
Sex						
Female	1.55 (1.52-1.58)	1.42 (1.38-1.45)	1.50 (1.43-1.58)	1.15 (1.12-1.19)	1.86 (1.81-1.91)	1.80 (1.75-1.85)
Male	1.0 [Reference]	1.0 [Reference]	1.0 [Reference]	1.0 [Reference]	1.0 [Reference]	1.0 [Reference]
Age group, y						
18-34	1.0 [Reference]	1.0 [Reference]	1.0 [Reference]	1.0 [Reference]	1.0 [Reference]	1.0 [Reference]
35-44	0.92 (0.88-0.95)	1.01 (0.96-1.05)	0.7 (0.64-0.76)	1.04 (0.98-1.10)	1.07 (1.02-1.13)	0.78 (0.75-0.82)
45-54	0.78 (0.75-0.80)	0.93 (0.89-0.97)	0.61 (0.57-0.67)	1.04 (0.99-1.11)	1.07 (1.02-1.12)	0.55 (0.53-0.58)
55-64	0.59 (0.57-0.61)	0.75 (0.72-0.78)	0.55 (0.51-0.60)	1.01 (0.96-1.07)	0.79 (0.75-0.83)	0.36 (0.34-0.37)
65-74	0.48 (0.46-0.49)	0.58 (0.55-0.60)	0.49 (0.46-0.54)	0.95 (0.89-1.00)	0.69 (0.66-0.73)	0.23 (0.22-0.24)
≥75	0.49 (0.46-0.52)	0.67 (0.62-0.71)	0.66 (0.58-0.74)	1.03 (0.95-1.12)	0.73 (0.68-0.79)	0.16 (0.14-0.17)
Race and ethnicity						
American Indian or Alaska Native	1.46 (1.23-1.73)	1.48 (1.22-1.80)	1.97 (1.43-2.69)	1.88 (1.52-2.33)	1.54 (1.25-1.9)	1.45 (1.16-1.81)
Asian	0.79 (0.73-0.84)	0.8 (0.73-0.88)	1.1 (0.94-1.30)	0.9 (0.80-1.01)	0.88 (0.79-0.97)	0.79 (0.72-0.87)
Black or African American	1.04 (0.97-1.11)	1.19 (1.09-1.29)	1.54 (1.33-1.77)	1.06 (0.95-1.18)	0.9 (0.82-1.00)	1.13 (1.03-1.24)
Hispanic	1.21 (1.14-1.28)	1.4 (1.31-1.50)	1.68 (1.49-1.89)	1.35 (1.24-1.46)	1.24 (1.15-1.34)	1.26 (1.17-1.36)
White	1.0 [Reference]	1.0 [Reference]	1.0 [Reference]	1.0 [Reference]	1.0 [Reference]	1.0 [Reference]
Multiracial or other^d^	1.22 (1.12-1.32)	1.3 (1.18-1.44)	1.51 (1.27-1.80)	1.24 (1.10-1.40)	1.21 (1.09-1.35)	1.26 (1.13-1.39)
Chronic health conditions						
None	1.0 [Reference]	1.0 [Reference]	1.0 [Reference]	1.0 [Reference]	1.0 [Reference]	1.0 [Reference]
1	1.47 (1.44-1.50)	1.59 (1.55-1.64)	1.62 (1.53-1.72)	1.40 (1.35-1.45)	1.29 (1.25-1.33)	1.55 (1.50-1.60)
2	2.04 (1.98-2.10)	2.37 (2.29-2.45)	2.57 (2.41-2.74)	1.83 (1.76-1.91)	1.66 (1.60-1.72)	2.17 (2.09-2.26)
3	2.87 (2.75-3.00)	3.47 (3.31-3.64)	3.99 (3.68-4.34)	2.53 (2.38-2.68)	2.23 (2.11-2.35)	2.95 (2.80-3.12)
≥4	4.03 (3.79-4.28)	5.09 (4.78-5.43)	6.28 (5.70-6.93)	3.71 (3.44-4.00)	3.12 (2.90-3.35)	4.07 (3.79-4.37)

Abbreviation: AOR, adjusted odds ratio.

^a^ This is a logistic regression model with long-term symptoms as the outcome and prior SARS-CoV-2 infection status as the main exposure. Covariates include sex, age category, race and ethnicity, and number of underlying medical conditions. State of residence was included as a random intercept. A total of 238 828 observations were included in the model with multiple imputation used for missing values.

^b^ Neurologic symptoms include fatigue, headache, malaise, difficulty sleeping, difficulty speaking, problems with balance, numbness, difficulty thinking or concentrating, dizziness, and difficulty swallowing. Gastrointestinal symptoms include loss of appetite, constipation, diarrhea, vomiting, and abdominal pain. Respiratory and cardiac symptoms include congestion, cough, shortness of breath, palpitations, throat pain, and chest pain. Other symptoms include bruising, menstruation changes, chills, hair loss, joint swelling, joint pain, skin changes, weight loss, change in taste, and change in smell. Mental health symptoms include anxiety, depression, posttraumatic stress disorder, and mood changes.

^c^ SARS-CoV-2 infection was defined as antinucleocapsid antibody positive or self-report of confirmed infection since March 2020.

^d^ Other indicates any race or ethnicity not listed.

### General, Physical, and Mental Health Survey Questions

A total of 20 448 of 81 108 individuals (25.2%) with SARS-CoV-2 infection and 39 029 of 151 794 (25.7%) without SARS-CoV-2 infection reported excellent general health in the past 4 weeks before the survey (eTable 7 in Supplement 1). Physical health at the time of the survey, compared with before the COVID-19 pandemic, was reported to be worse by 13 413 of 81 096 (16.5%) of those with prior SARS-CoV-2 infection compared with 16 615 of 151 830 (10.9%) of those without. Similarly, mental health at the time of the survey, compared with before the COVID-19 pandemic, was reported as worse for 15 336 of 81 108 (18.9%) of those with prior SARS-CoV-2 compared with 22 781 of 151 810 (15.0%) of those without.

## Discussion

Using a large data set that paired serologic testing and self-reported SARS-CoV-2 infection, we found that 43.3% of individuals with prior SARS-CoV-2 infection reported onset of new long-term symptoms during the pandemic, which is within the range reported by 3 other studies (40%-55%)^12,13,14^; in comparison, 22.1% of those without SARS-CoV-2 infection reported long-term symptoms. The difference in these proportions suggests that 21.2% of donors with prior SARS-CoV-2 infection likely experienced long-term symptoms attributed to their infection. A lower estimate of long-term symptoms came from a meta-analysis that estimated that 6.2% of nonhospitalized individuals who had symptomatic COVID-19 infection experienced 1 of 3 post–COVID-19 condition symptom clusters with a duration of 4 months.^15^ The actual prevalence of long-term symptoms after COVID-19 is unclear due to varying definitions, study designs, and the lack of an uninfected control group.^13^ Understanding the true burden of post–COVID-19 conditions is critical, and inclusion of a control group as a reference for baseline levels of symptoms should be prioritized.

Because of the availability of serologic data on study participants, this study allowed examination of outcomes stratified by serologic and self-report status. Those who reported no prior infection but were serologically positive likely had asymptomatic or mild infections and were less likely to report long-term symptoms since March 2020 compared with those with reported confirmed infections (34.9% vs 54.0%; eTable 4 in Supplement 1). This finding is in line with a prior study showing an association between disease severity and the development of post–COVID-19 conditions.^16^ Importantly, the serologic data also allowed for differentiation among individuals who reported no prior infection, showing a higher prevalence of new long-term symptoms in those with anti-N antibodies and presumed mild or asymptomatic infections compared with the control participants without anti-N antibodies (34.9% vs 22.1%). Differences in results from this analysis compared with previous studies may be related to differences in accuracy of assays and the combination of serologic evidence and self-reported, laboratory-confirmed history used.^8,10^

This study informs our understanding of the mental health consequences resulting from the COVID-19 pandemic.^17^ The pandemic has been associated with increased rates of anxiety, depression, and other mental health disorders, even among those who were not infected.^18,19^ Factors contributing to these negative mental health outcomes include social isolation, financial strain, disrupted routines and schedules, and the fear and uncertainty surrounding the pandemic.^18^ We examined mental health markers by measuring symptoms in those with and without SARS-CoV-2 infection. Of all the long-term symptoms, the percentages of participants having long-term anxiety, depression, posttraumatic stress disorder, and mood changes were more similar in the uninfected control group compared with the infected groups, and logistic regression analysis confirmed the minimal difference in mental health symptoms after adjusting for other covariates. These findings suggest that these symptoms may be less attributable to the SARS-CoV-2 infection process itself and more to the COVID-19 pandemic having an impact on mental health across society during the pandemic. Overall, an appreciable number of participants reported having significantly worse mental health compared with before the COVID-19 pandemic, ranging from 15.0% among uninfected participants to 18.9% among participants with infection. Notably, individuals in both the infected and uninfected groups were more likely to report worsened mental health than worsened physical health and conversely were less likely to report improved mental health than improved physical health compared with before the COVID-19 pandemic. These data underscore the importance of considering mental health in infectious disease public health responses.

In this study, older age was not associated with increased risk of new long-term symptoms, including long-term respiratory or cardiac symptoms. Although many studies have shown that older age is associated with post–COVID-19 conditions,^20,21^ another study also observed a decreasing risk at older ages,^22^ perhaps resulting from lower symptom reporting in older adults and mortality bias. These biases may also be present in this blood donor population.

### Limitations

This study is subject to several limitations. The American Red Cross donor population is not demographically representative of the US population, and those experiencing severe or debilitating post–COVID-19 conditions would be unlikely to donate blood, which limits external validity. In addition, those with SARS-CoV-2 infection and who experienced long-term symptoms may have been more likely to respond to the survey. Because donors were asked about new long-term symptoms in the survey, this study does not capture worsening of underlying conditions. Serologic data may have only been collected several months or longer before survey administration, which could lead to an underestimation of the number of individuals with SARS-CoV-2 infections. The ability of serologic testing to detect prior infection varies widely depending on assay used, with the assays used in the current study showing durability for at least 1 year after infection.^10^ Additionally, the high-sensitivity anti-N assay used in this analysis detects only natural infection and not vaccine-derived immunity because the N antigen is not present in COVID-19 vaccines to date. Reinfection and severity of COVID-19 infection were not included in this analysis but may impact the association with long-term symptoms. Recall error on the presence of new long-term symptoms since March 2020 (approximately 2 years prior) is a potential limitation but presumably nondifferential among those with and without prior SARS-CoV-2 infection. Because data on long-term symptom start and end dates were not collected, it was not possible to know when symptoms started in relation to SARS-CoV-2 infection. Therefore, the long-term symptoms reported by participants may have occurred before SARS-CoV-2 infection from other ailments. However, we included the SARS-CoV-2 uninfected comparison group to estimate a baseline prevalence of those symptoms. Additionally, if participants had onset of new long-term symptoms before SARS-CoV-2 infection, this would bias the ORs toward the null.

## Conclusions

This study found that long-term symptoms lasting more than 4 weeks were common in the adult population, but there was a significantly higher prevalence among those with SARS-CoV-2 infection. Mental health symptoms occurred almost as often in both those with and without prior SARS-CoV-2 infection, suggesting the presence of indirect effects. Continued efforts are needed to define and track long-term sequelae of SARS-CoV-2 using a control group without infection and serologic information to include those who had asymptomatic or unidentified infections.

## Notes

Supplement 1. **eFigure.** Study timeline including months of serologic testing and survey administration **eTable 1.** List and categories of long-term symptoms included in survey questions **eTable 2.** Comparison of demographic factors among survey responders, non-responders, and the full ARC population **eTable 3.** Demographics by SARS-CoV-2 prior infection status among blood donors surveyed between February and April 2022 **eTable 4.** Number and types of long-term symptoms by serologic and self-reported SARS-CoV-2 status **eTable 5.** Demographic features, underlying conditions, characteristics of acute illness among individuals with new symptoms lasting >4 weeks since March 2020 and history of SARS-CoV-2 infection, by symptom category **eTable 6.** Number and proportion of new symptoms (lasting >4 weeks since March 2020) among those with a history of SARS-CoV-2 that are resolved and ongoing at the time of survey **eTable 7.** Physical and mental health in the last 4 weeks among American Red Cross blood donors surveyed during February and April 2022 **eAppendix.** Survey

Supplement 2.
Data Sharing Statement
