# Supplementary material for: Long-Term Symptoms Associated With SARS-CoV-2 Infection Among Blood Donors
Source: JAMA Netw Open. 2024 Apr 8;7(4):e245611. doi: 10.1001/jamanetworkopen.2024.5611 (PMC11002700; doi:10.1001/jamanetworkopen.2024.5611)
Supplement: Supplement 1. — eFigure. Study timeline including months of serologic testing and survey administration eTable 1. List and categories of long-term symptoms included in survey questions eTable 2. Comparison of demographic factors among survey responders, non-responders, and the full ARC population eTable 3. Demographics by SARS-CoV-2 prior infection status among blood donors surveyed between February and April 2022 eTable 4. Number and types of long-term symptoms by serologic and self-reported SARS-CoV-2 status eTable 5. Demographic features, underlying conditions, characteristics of acute illness among individuals with new symptoms lasting >4 weeks since March 2020 and history of SARS-CoV-2 infection, by symptom category eTable 6. Number and proportion of new symptoms (lasting >4 weeks since March 2020) among those with a history of SARS-CoV-2 that are resolved and ongoing at the time of survey eTable 7. Physical and mental health in the last 4 weeks among American Red Cross blood donors surveyed during February and April 2022 eAppendix. Survey [file jamanetwopen-e245611-s001.pdf]

Supplemental Online Content

Shah MM, Spencer BR, James-Gist J, et al. Long-term symptoms associated with SARS-CoV-2 infection among blood donors. *JAMA Netw Open*. 2024;7(4):e245611. doi:10.1001/jamanetworkopen.2024.5611

eFigure 1. Study timeline including months of serologic testing and survey administration
eTable 1. List and categories of long-term symptoms included in survey questions
eTable 2. Comparison of demographic factors among survey responders, non-responders, and the full American Red Cross population
eTable 3. Demographics by SARS-CoV-2 prior infection status among blood donors surveyed between February 22, 2022 – April 21, 2022
eTable 4. Number and types of long-term symptoms by serologic and self-reported SARS-CoV-2 status
eTable 5. Demographic features, underlying conditions, characteristics of acute illness among individuals with new symptoms lasting >4 weeks since March 2020 and history of SARS-CoV-2 infection, by symptom category
eTable 6. Number and proportion of new symptoms (lasting >4 weeks since March 2020) among those with a history of SARS-CoV-2 that are resolved and ongoing at the time of survey
eTable 7. Physical and mental health in the last 4 weeks among American Red Cross blood donors surveyed during February 22, 2022 – April 21, 2022
eAppendix. Survey

This supplemental material has been provided by the authors to give readers additional information about their work.

© 2024 Shah MM et al. *JAMA Network Open.*

eFigure 1: Study timeline including months of serologic testing and survey administration

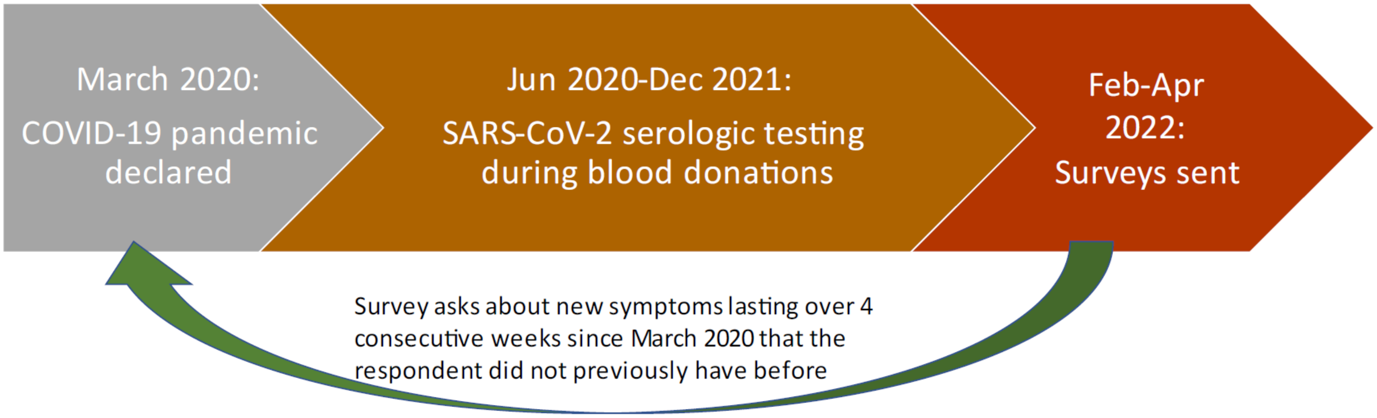

© 2024 Shah MM et al. *JAMA Network Open.*

eTable 1: List and categories of long-term symptoms included in survey questions

**Neurologic**	Fatigue/weakness
	Headache
	Symptoms that get worse after physical or mental activities (post-exertional malaise)
	Problems sleeping
	Problems speaking or communicating
	Problems with balance/movement
	Tingling/numbness in any part of body
	Difficulty thinking or concentrating (sometimes referred to as “brain fog”)
	Dizziness/lightheadedness/fainting
	Problems swallowing or chewing
**Gastrointestinal**	Appetite changes
	Constipation
	Diarrhea
	Nausea/Vomiting
	Stomach Pain
**Cardiac and Respiratory**	Congestion/runny nose
	Cough
	Shortness of breath
	Palpitations (heart racing or pounding)
	Sore throat
	Chest pain/pressure
**Other**	Bruising/bleeding easily
	Change in menstruation cycle
	Fever or chills
	Hair loss
	Joint swelling
	Joint/muscle pain
	Skin changes
	Unintentional weight loss
	Change in taste
	Change in smell
**Mental health**	Anxiety
	Depression
	Post-traumatic stress disorder
	Change in mood

© 2024 Shah MM et al. *JAMA Network Open.*

eTable 2: Comparison of demographic factors among survey responders, non-responders, and the full American Red Cross population

		Survey Responders, No (%) N=272,965	Survey Non-Responders, No (%) N=545,396	Full ARC Population, No (%) [a] N=3,002,104	US Population 2020, No (%) N=253,272,570
**Sex**	Female	158043 (57.9)	295,890 (54.3)	1,699,982 (56.6)	129,910,794 (51.3)
	Male	114922 (42.1)	249,506 (45.8)	1,302,122 (43.4)	123,361,776 (48.7)
**Age category** [b]	18-34 years	24600 (9.0)	112991 (20.7)	709,421 (23.6)	75,920,901 (30.0)
	35-54 years	84297 (30.9)	193279 (35.4)	1038056 (34.6)	82,887,413 (32.7)
	55-74 years	149249 (54.7)	218466 (40.1)	1051887 (35.0)	72,649,389 (28.7)
	≥75 years	14819 (5.4)	20660 (3.8)	97872 (3.3)	21,814,867 (8.6)
**Race and ethnicity** [c]	Asian	6090 (2.2)	17502 (3.2)	86644 (2.9)	19,618,719 (5.9)
	American Indian	672 (0.2)	1770 (0.3)	9652 (0.3)	2,251,699 (0.7)
	Hispanic	7031 (2.6)	27462 (5.0)	136739 (4.6)	62,080,044 (18.7)
	Non-Hispanic Black	5150 (1.9)	16302 (2.9)	88111 (2.9)	39,940,338 (12.1)
	Non-Hispanic White	250201 (91.7)	470370 (86.2)	2613083 (87.0)	191,697,647 (57.8)
	Other	3291 (1.2)	10437 (1.9)	56442 (1.9)	15,860,834 (4.8)
	missing	530 (0.2)	1553 (0.3)	11433 (0.4)	-
**US Census region**[d]	Midwest	91402 (33.5)	169973 (31.2)	1030163 (34.3)	53,512,051 (20.7)
	West	57406 (21.0)	124626 (22.9)	586970 (19.6)	61,028,132 (23.6)
	South	65017 (23.8)	143899 (26.4)	801113 (26.7)	97,904,314 (37.9)
	Northeast	58937 (21.6)	106372 (19.5)	579300 (19.3)	45,898,784 (17.8)
	missing	203 (0.1)	526 (0.1)	4558 (0.2)	-
**Vaccination status at last donation before 12/31/21**	Not Vaccinated	39795 (14.6)	122091 (22.4)	837271 (27.9)	-
	Vaccinated	231693 (84.9)	413624 (75.8)	1577271 (52.5)	-
	missing	1477 (0.5)	9681 (1.8)	587562 (19.6)	-
**Serological testing**	(% N positive) at the last donation with serology available	56407 (20.7)	145,705 (26.7)	384522 (12.8)	-
	Median date of those with N-positive donation	03/11/2021	03/20/2021	03/18/2021	-

[a] 3.5% of unique donors in the ARC population were under 18 years of age and excluded from the study.
[b] United States Census Bureau ACS 2020, 18 years of age and older, data.census.gov/cedsci/table?d=ACS%205-Year%20Estimates%20Detailed%20Tables, data.census.gov/cedsci/table?q=demographics&tid=ACSDP5Y2020.DP05
[c] Race ethnicity distribution for the US population includes all ages. For non-Hispanic strata, reflects all ages (not only >18) due to availability of data. 2020 Census Redistricting: Supplementary Tables

© 2024 Shah MM et al. *JAMA Network Open.*

eTable 3: Demographics by SARS-CoV-2 prior infection status among blood donors surveyed during February 22, 2022 – April 21, 2022

		SARS-CoV-2 Infection (anti-N antibody-positive or self-report), No (%) N=83,015	95% CI	No SARS-CoV-2 Infection, No (%) N=154,283	95% CI
**Sex**	Female	49,920 / 83,015 (60.1%)	59.7-60.3	87,864 / 154,283 (56.9%)	56.7-57.2
	Male	33,095 / 83,015 (39.9%)	39.7-40.3	66,419 / 154,283 (43.1%)	42.8-43.3
**Age category**	18-34 years	9,629 / 83,015 (11.6%)	11.4-11.8	10,154 / 154,283 (6.6%)	6.5-6.7
	35-44 years	14,228 / 83,015 (17.1%)	16.8-17.3	16,237 / 154,283 (10.5%)	10.4-10.7
	45-54 years	18,233 / 83,015 (22.0%)	21.7-22.2	23,432 / 154,283 (15.2%)	15.0-15.4
	55-64 years	23,540 / 83,015 (28.4%)	28.1-28.7	44,985 / 154,283 (29.2%)	28.9-29.4
	65-74 years	14,963 / 83,015 (18.0%)	17.8-18.4	48,572 / 154,283 (31.5%)	31.3-31.7
	>74 years	2,422 / 83,015 (2.9%)	2.8-3.0	10,903 / 154,283 (7.1%)	6.9-7.2
**Race and ethnicity** [a]	American Indian or Alaska Native	251 / 82,861 (0.3%)	0.3-0.3	332 / 153,995 (0.2%)	0.2-0.2
	Asian	1,276 / 82,861 (1.5%)	1.5-1.6	3,648 / 153,995 (2.4%)	2.3-2.4
	Black or African American	1,407 / 82,861 (1.7%)	1.6-1.8	2,900 / 153,995 (1.9%)	1.8-2.0
	Hispanic	2,590 / 82,861 (3.1%)	3.0-3.2	3,121 / 153,995 (2.0%)	2.0-2.1
	White	76,389 / 82,861 (92.2%)	92.0-92.4	142,179 / 153,995 (92.3%)	92.2-92.5
	Multiracial and other	948 / 82,861 (1.1%)	1.1-1.2	1,815 / 153,995 (1.2%)	1.1-1.2
	missing	154		288	
**Chronic health conditions** [b]	≥1 Chronic health conditions	35,059 / 82,137 (42.7%)	42.3-43.0	72,689 / 152,990 (47.5%)	47.3-47.8
	Missing	878		1,293	
**US Census region**	Midwest	32,366 / 82,948 (39.0%)	38.7-39.4	47,580 / 154,180 (30.9%)	30.6-31.1
	Northeast	14,838 / 82,948 (17.9%)	17.6-18.2	36,397 / 154,180 (23.6%)	23.4-23.8
	South	19,703 / 82,948 (23.8%)	23.5-24.0	36,530 / 154,180 (23.7%)	23.5-23.9
	West	16,041 / 82,948 (19.3%)	19.1-19.6	33,673 / 154,180 (21.8%)	21.6-22.0
	missing	67		103	
**SARS-CoV-2 vaccination status**	1 or more vaccine dose	66,111 / 82,538 (80.1%)	79.8-80.4	147,851 / 153,891 (96.1%)	96.0-96.2
	Unvaccinated	16,427 / 82,538 (19.9%)	19.6-20.2	6,040 / 153,891 (3.9%)	3.8-4.0
	missing	477		392	

[a] Race and ethnicity data are routinely collected at blood donation. Missing indicates any race or ethnicity not otherwise listed.
[b] Chronic health conditions include chronic pain, anxiety, depression, chronic headaches, stroke, kidney disease, liver disease, heart disease, lung disease, high blood pressure, diabetes, immune disorder, or cancer.

© 2024 Shah MM et al. *JAMA Network Open.*

eTable 4: Number and types of long-term symptoms by serologic and self-reported SARS-CoV-2 status

	Reported infection [a], No (%) N = 59,006 [b]	Anti-N antibody positive and reported infection, No (%) N = 26,103	Anti-N antibody positive and no reported infection, No (%) N = 23,979	Perpetual Anti-N antibody negative and reported infection [c], No (%) N = 5,654
**Any persistent Symptoms**	27,537 / 59,006 (46.7%)	14,089 / 26,103 (54.0%)	8,377 / 23,979 (34.9%)	2,920 / 5,654 (51.6%)
**Mean number of symptoms among those with any symptoms**	3.14	3.28	2.80	3.40
**Neurologic symptoms**	14,924 / 57,003 (26.2%)	8,040 / 25,288 (31.8%)	4,017 / 23,187 (17.3%)	1,585 / 5,463 (29.0%)
Fatigue	7,155 / 57,003 (12.6%)	4,019 / 25,288 (15.9%)	1,720 / 23,187 (7.4%)	746 / 5,463 (13.7%)
Headache	2,709 / 57,003 (4.8%)	1,481 / 25,288 (5.9%)	679 / 23,187 (2.9%)	284 / 5,463 (5.2%)
Post-exertional malaise	1,690 / 57,003 (3.0%)	933 / 25,288 (3.7%)	334 / 23,187 (1.4%)	213 / 5,463 (3.9%)
Difficulty sleeping	4,324 / 57,003 (7.6%)	2,171 / 25,288 (8.6%)	1,204 / 23,187 (5.2%)	474 / 5,463 (8.7%)
Difficulty speaking	1,415 / 57,003 (2.5%)	837 / 25,288 (3.3%)	297 / 23,187 (1.3%)	163 / 5,463 (3.0%)
Problems with balance	1,663 / 57,003 (2.9%)	950 / 25,288 (3.8%)	444 / 23,187 (1.9%)	209 / 5,463 (3.8%)
Numbness	2,245 / 57,003 (3.9%)	1,274 / 25,288 (5.0%)	735 / 23,187 (3.2%)	259 / 5,463 (4.7%)
Difficulty thinking or concentrating	8,166 / 57,003 (14.3%)	4,688 / 25,288 (18.5%)	1,979 / 23,187 (8.5%)	912 / 5,463 (16.7%)
Dizziness	1,937 / 57,003 (3.4%)	1,086 / 25,288 (4.3%)	494 / 23,187 (2.1%)	214 / 5,463 (3.9%)
Difficulty swallowing	454 / 57,003 (0.8%)	263 / 25,288 (1.0%)	125 / 23,187 (0.5%)	56 / 5,463 (1.0%)
**Gastrointestinal symptoms**	2,861 / 56,959 (5.0%)	1,509 / 25,123 (6.0%)	800 / 23,190 (3.4%)	342 / 5,460 (6.3%)
Loss of appetite	1,360 / 56,959 (2.4%)	757 / 25,123 (3.0%)	356 / 23,190 (1.5%)	166 / 5,460 (3.0%)
Constipation	694 / 56,959 (1.2%)	368 / 25,123 (1.5%)	207 / 23,190 (0.9%)	73 / 5,460 (1.3%)
Diarrhea	799 / 56,959 (1.4%)	424 / 25,123 (1.7%)	218 / 23,190 (0.9%)	96 / 5,460 (1.8%)
Vomiting	396 / 56,959 (0.7%)	209 / 25,123 (0.8%)	86 / 23,190 (0.4%)	53 / 5,460 (1.0%)
Abdominal pain	703 / 56,959 (1.2%)	383 / 25,123 (1.5%)	222 / 23,190 (1.0%)	81 / 5,460 (1.5%)
**Respiratory/cardiac symptoms**	9,999 / 56,915 (17.6%)	4,999 / 25,154 (19.9%)	2,636 / 23,123 (11.4%)	1,039 / 5,457 (19.0%)
Congestion	3,340 / 56,915 (5.9%)	1,468 / 25,154 (5.8%)	987 / 23,123 (4.3%)	354 / 5,457 (6.5%)
Cough	4,292 / 56,915 (7.5%)	1,827 / 25,154 (7.3%)	1,007 / 23,123 (4.4%)	450 / 5,457 (8.2%)
Shortness of breath	3,435 / 56,915 (6.0%)	2,104 / 25,154 (8.4%)	836 / 23,123 (3.6%)	362 / 5,457 (6.6%)
Palpitations	2,052 / 56,915 (3.6%)	1,220 / 25,154 (4.9%)	528 / 23,123 (2.3%)	215 / 5,457 (3.9%)
Throat pain	756 / 56,915 (1.3%)	310 / 25,154 (1.2%)	212 / 23,123 (0.9%)	76 / 5,457 (1.4%)
Chest pain	1,328 / 56,915 (2.3%)	769 / 25,154 (3.1%)	310 / 23,123 (1.3%)	144 / 5,457 (2.6%)
**Other symptoms**	14,106 / 56,987 (24.8%)	8,307 / 25,246 (32.9%)	4,412 / 23,227 (19.0%)	1,658 / 5,472 (30.3%)
Bruising	525 / 56,987 (0.9%)	279 / 25,246 (1.1%)	130 / 23,227 (0.6%)	87 / 5,472 (1.6%)
Menstruation changes [d]	1,543 / 34,979 (4.4%)	690 / 15,498 (4.5%)	374 / 13,188 (2.8%)	160 / 3,304 (4.8%)
Chills	370 / 56,987 (0.6%)	201 / 25,246 (0.8%)	130 / 23,227 (0.6%)	42 / 5,472 (0.8%)
Hair loss	3,530 / 56,987 (6.2%)	2,129 / 25,246 (8.4%)	897 / 23,227 (3.9%)	399 / 5,472 (7.3%)
Joint swelling	888 / 56,987 (1.6%)	534 / 25,246 (2.1%)	284 / 23,227 (1.2%)	100 / 5,472 (1.8%)
Joint pain	3,593 / 56,987 (6.3%)	2,083 / 25,246 (8.3%)	1,111 / 23,227 (4.8%)	415 / 5,472 (7.6%)
Skin changes	961 / 56,987 (1.7%)	546 / 25,246 (2.2%)	256 / 23,227 (1.1%)	99 / 5,472 (1.8%)
Weight loss	633 / 56,987 (1.1%)	348 / 25,246 (1.4%)	133 / 23,227 (0.6%)	80 / 5,472 (1.5%)
Change in taste	5,887 / 56,987 (10.3%)	3,803 / 25,246 (15.1%)	1,848 / 23,227 (8.0%)	729 / 5,472 (13.3%)
Change in smell	6,618 / 56,987 (11.6%)	4,323 / 25,246 (17.1%)	2,176 / 23,227 (9.4%)	809 / 5,472 (14.8%)
**Mental health symptoms**	7,565 / 57,281 (13.2%)	3,510 / 25,319 (13.9%)	2,066 / 23,329 (8.9%)	797 / 5,498 (14.5%)
Anxiety	4,749 / 57,281 (8.3%)	2,203 / 25,319 (8.7%)	1,292 / 23,329 (5.5%)	496 / 5,498 (9.0%)
Depression	3,339 / 57,281 (5.8%)	1,547 / 25,319 (6.1%)	879 / 23,329 (3.8%)	371 / 5,498 (6.7%)
Post-traumatic stress disorder	672 / 57,281 (1.2%)	352 / 25,319 (1.4%)	179 / 23,329 (0.8%)	88 / 5,498 (1.6%)
Mood changes	3,601 / 57,281 (6.3%)	1,659 / 25,319 (6.6%)	965 / 23,329 (4.1%)	403 / 5,498 (7.3%)

[a] Includes individuals who report a swab-confirmed infection or healthcare provider diagnosis for acute SARS-CoV-2 infection with or without anti-nucleocapsid antibody positivity.
[b] n / N (%)
[c] Includes individuals who provide a confirmed infection date but on serologic testing 14-365 days after infection and who are anti-nucleocapsid antibody negative.
[d] This row of denominators only includes females who reported at least one long-term symptom.

© 2024 Shah MM et al. *JAMA Network Open.*

eTable 5: Demographic features, underlying conditions, characteristics of acute illness among individuals[a] with new symptoms lasting >4 weeks since March 2020 and history of SARS-CoV-2 infection, by symptom category

		Any neurologic symptom, No (%) N=18,949	Any gastrointestinal symptom, No (%) N=3,664	Any respiratory/cardiac symptom, No (%) N=12,639	Any "other" symptom, No (%) N=18,525	Any mental health symptom, No (%) N=9,634
**Sex**	Female	12,952 / 18,949 (68.4%)	2,616 / 3,664 (71.4%)	8,179 / 12,639 (64.7%)	13,475 / 18,525 (72.7%)	7,156 / 9,634 (74.3%)
	Male	5,997 / 18,949 (31.6%)	1,048 / 3,664 (28.6%)	4,460 / 12,639 (35.3%)	5,050 / 18,525 (27.3%)	2,478 / 9,634 (25.7%)
**Age category**	18-34 years	2,360 / 18,949 (12.5%)	639 / 3,664 (17.4%)	1,411 / 12,639 (11.2%)	2,276 / 18,525 (12.3%)	1,942 / 9,634 (20.2%)
	35-44 years	3,618 / 18,949 (19.1%)	640 / 3,664 (17.5%)	2,191 / 12,639 (17.3%)	3,622 / 18,525 (19.6%)	2,365 / 9,634 (24.5%)
	45-54 years	4,562 / 18,949 (24.1%)	828 / 3,664 (22.6%)	2,945 / 12,639 (23.3%)	4,660 / 18,525 (25.2%)	2,346 / 9,634 (24.4%)
	55-64 years	5,178 / 18,949 (27.3%)	906 / 3,664 (24.7%)	3,572 / 12,639 (28.3%)	4,951 / 18,525 (26.7%)	2,025 / 9,634 (21.0%)
	65-74 years	2,760 / 18,949 (14.6%)	544 / 3,664 (14.8%)	2,148 / 12,639 (17.0%)	2,641 / 18,525 (14.3%)	849 / 9,634 (8.8%)
	>74 years	471 / 18,949 (2.5%)	107 / 3,664 (2.9%)	372 / 12,639 (2.9%)	375 / 18,525 (2.0%)	107 / 9,634 (1.1%)
**Race and ethnicity** [b]	American Indian	92 / 18,912 (0.5%)	23 / 3,654 (0.6%)	70 / 12,616 (0.6%)	90 / 18,489 (0.5%)	42 / 9,610 (0.4%)
	Asian	239 / 18,912 (1.3%)	57 / 3,654 (1.6%)	173 / 12,616 (1.4%)	221 / 18,489 (1.2%)	172 / 9,610 (1.8%)
	Black	392 / 18,912 (2.1%)	112 / 3,654 (3.1%)	233 / 12,616 (1.8%)	270 / 18,489 (1.5%)	227 / 9,610 (2.4%)
	Hispanic	824 / 18,912 (4.4%)	211 / 3,654 (5.8%)	505 / 12,616 (4.0%)	733 / 18,489 (4.0%)	498 / 9,610 (5.2%)
	White	17,093 / 18,912 (90.4%)	3,178 / 3,654 (87.0%)	11,469 / 12,616 (90.9%)	16,925 / 18,489 (91.5%)	8,500 / 9,610 (88.4%)
	Other	272 / 18,912 (1.4%)	73 / 3,654 (2.0%)	166 / 12,616 (1.3%)	250 / 18,489 (1.4%)	171 / 9,610 (1.8%)
	missing	37	10	23	36	24
**Chronic health conditions** [c]	≥1 chronic health conditions	10,355 / 18,725 (55.3%)	2,199 / 3,622 (60.7%)	6,683 / 12,496 (53.5%)	8,997 / 18,317 (49.1%)	5,117 / 9,510 (53.8%)
	Missing	224	42	143	208	125
**For any of the long-term symptoms, did you ever see a health care professional?**	Yes	7,118 / 18,561 (38.3%)	1,830 / 3,580 (51.1%)	5,556 / 12,409 (44.8%)	6,028 / 18,174 (33.2%)	4,236 / 9,349 (45.3%)
	No	11,116 / 18,561 (59.9%)	1,682 / 3,580 (47.0%)	6,665 / 12,409 (53.7%)	11,839 / 18,174 (65.1%)	4,908 / 9,349 (52.5%)
	Unsure	327 / 18,561 (1.8%)	68 / 3,580 (1.9%)	188 / 12,409 (1.5%)	307 / 18,174 (1.7%)	205 / 9,349 (2.2%)
	Missing	388	84	230	351	285
**Attribute long-term symptoms to COVID-19**	Yes	9,891 / 18,521 (53.4%)	1,913 / 3,577 (53.5%)	7,153 / 12,387 (57.7%)	11,407 / 18,119 (63.0%)	3,728 / 9,320 (40.0%)
	No	3,922 / 18,521 (21.2%)	799 / 3,577 (22.3%)	2,265 / 12,387 (18.3%)	3,076 / 18,119 (17.0%)	3,641 / 9,320 (39.1%)
	Unsure	4,708 / 18,521 (25.4%)	865 / 3,577 (24.2%)	2,969 / 12,387 (24.0%)	3,636 / 18,119 (20.1%)	1,951 / 9,320 (20.9%)
	Missing	428	87	252	406	314
**After your first COVID-19 infection, how long did it take to return to your usual health?**	<1 month	4,383 / 15,197 (28.8%)	743 / 2,858 (26.0%)	2,904 / 10,091 (28.8%)	5,703 / 14,924 (38.2%)	2,852 / 7,404 (38.5%)
	1-2 months	4,070 / 15,197 (26.8%)	672 / 2,858 (23.5%)	2,632 / 10,091 (26.1%)	3,683 / 14,924 (24.7%)	1,676 / 7,404 (22.6%)
	3-5 months	2,119 / 15,197 (13.9%)	379 / 2,858 (13.3%)	1,331 / 10,091 (13.2%)	1,749 / 14,924 (11.7%)	760 / 7,404 (10.3%)
	6-12 months	1,163 / 15,197 (7.7%)	201 / 2,858 (7.0%)	749 / 10,091 (7.4%)	948 / 14,924 (6.4%)	465 / 7,404 (6.3%)
	>12 months	300 / 15,197 (2.0%)	65 / 2,858 (2.3%)	198 / 10,091 (2.0%)	259 / 14,924 (1.7%)	142 / 7,404 (1.9%)
	Never returned to usual health	2,531 / 15,197 (16.7%)	690 / 2,858 (24.1%)	1,916 / 10,091 (19.0%)	2,113 / 14,924 (14.2%)	1,241 / 7,404 (16.8%)
	Unsure	631 / 15,197 (4.2%)	108 / 2,858 (3.8%)	361 / 10,091 (3.6%)	469 / 14,924 (3.1%)	268 / 7,404 (3.6%)
	Missing	3,752	806	2,548	3,601	2,230

[a] Individuals with long-term symptoms in multiple categories are included in the totals for each category of symptoms.
[b] Race and ethnicity data are routinely collected at blood donation. Missing indicates any race or ethnicity not otherwise listed.
[c] Chronic health conditions include chronic pain, anxiety, depression, chronic headaches, stroke, kidney disease, liver disease, heart disease, lung disease, high blood pressure, diabetes, immune disorder, or cancer.

© 2024 Shah MM et al. *JAMA Network Open.*

eTable 6: Number and proportion of new symptoms (lasting >4 weeks since March 2020) among those with a history of SARS-CoV-2 that are resolved and ongoing at the time of survey

	**Total number reported by all participants, No (%)**	**Total reported as resolved, No (%)**	**Total reported as ongoing, No (%)**
**Long-term symptom** [a]	N = 89,049	N = 13,799	N = 75,250
Difficulty thinking or concentrating	9,695 / 89,049 (10.9%)	1,611 / 13,799 (11.7%)	8,084 / 75,250 (10.7%)
Fatigue	8,484 / 89,049 (9.5%)	1,682 / 13,799 (12.2%)	6,802 / 75,250 (9.0%)
Difficulty sleeping	5,283 / 89,049 (5.9%)	454 / 13,799 (3.3%)	4,829 / 75,250 (6.4%)
Cough	5,038 / 89,049 (5.7%)	1,594 / 13,799 (11.6%)	3,444 / 75,250 (4.6%)
Change in smell	4,240 / 89,049 (4.8%)	889 / 13,799 (6.4%)	3,351 / 75,250 (4.5%)
Congestion	4,153 / 89,049 (4.7%)	832 / 13,799 (6.0%)	3,321 / 75,250 (4.4%)
Shortness of breath	4,106 / 89,049 (4.6%)	715 / 13,799 (5.2%)	3,391 / 75,250 (4.5%)
Change in taste	4,054 / 89,049 (4.6%)	765 / 13,799 (5.5%)	3,289 / 75,250 (4.4%)
Anxiety	3,799 / 89,049 (4.3%)	503 / 13,799 (3.6%)	3,296 / 75,250 (4.4%)
Joint pain	3,467 / 89,049 (3.9%)	357 / 13,799 (2.6%)	3,110 / 75,250 (4.1%)
Headache	3,195 / 89,049 (3.6%)	443 / 13,799 (3.2%)	2,752 / 75,250 (3.7%)
Change in mood	3,029 / 89,049 (3.4%)	364 / 13,799 (2.6%)	2,665 / 75,250 (3.5%)
Numbness	2,849 / 89,049 (3.2%)	204 / 13,799 (1.5%)	2,645 / 75,250 (3.5%)
Hair loss	2,847 / 89,049 (3.2%)	443 / 13,799 (3.2%)	2,404 / 75,250 (3.2%)
Depression	2,710 / 89,049 (3.0%)	328 / 13,799 (2.4%)	2,382 / 75,250 (3.2%)
Palpitations	2,501 / 89,049 (2.8%)	357 / 13,799 (2.6%)	2,144 / 75,250 (2.8%)
Dizziness	2,292 / 89,049 (2.6%)	289 / 13,799 (2.1%)	2,003 / 75,250 (2.7%)
Problems with balance	1,996 / 89,049 (2.2%)	152 / 13,799 (1.1%)	1,844 / 75,250 (2.5%)
Post-exertional malaise	1,948 / 89,049 (2.2%)	179 / 13,799 (1.3%)	1,769 / 75,250 (2.4%)
Problems speaking	1,643 / 89,049 (1.8%)	108 / 13,799 (0.8%)	1,535 / 75,250 (2.0%)
Chest pain	1,541 / 89,049 (1.7%)	253 / 13,799 (1.8%)	1,288 / 75,250 (1.7%)
Appetite changes	1,308 / 89,049 (1.5%)	154 / 13,799 (1.1%)	1,154 / 75,250 (1.5%)
Menstruation changes [b]	1,177 / 89,049 (1.3%)	194 / 13,799 (1.4%)	983 / 75,250 (1.3%)
Joint swelling	927 / 89,049 (1.0%)	69 / 13,799 (0.5%)	858 / 75,250 (1.1%)
Sore throat	874 / 89,049 (1.0%)	265 / 13,799 (1.9%)	609 / 75,250 (0.8%)
Skin changes	823 / 89,049 (0.9%)	86 / 13,799 (0.6%)	737 / 75,250 (1.0%)
Abdominal pain	705 / 89,049 (0.8%)	58 / 13,799 (0.4%)	647 / 75,250 (0.9%)
Diarrhea	685 / 89,049 (0.8%)	83 / 13,799 (0.6%)	602 / 75,250 (0.8%)
Post-traumatic stress disorder	655 / 89,049 (0.7%)	47 / 13,799 (0.3%)	608 / 75,250 (0.8%)
Constipation	624 / 89,049 (0.7%)	49 / 13,799 (0.4%)	575 / 75,250 (0.8%)
Bruising	556 / 89,049 (0.6%)	39 / 13,799 (0.3%)	517 / 75,250 (0.7%)
Problems swallowing	555 / 89,049 (0.6%)	35 / 13,799 (0.3%)	520 / 75,250 (0.7%)
Weight loss	525 / 89,049 (0.6%)	99 / 13,799 (0.7%)	426 / 75,250 (0.6%)
Nausea/vomiting	384 / 89,049 (0.4%)	45 / 13,799 (0.3%)	339 / 75,250 (0.5%)
Chills	381 / 89,049 (0.4%)	54 / 13,799 (0.4%)	327 / 75,250 (0.4%)

[a] Surveys were conducted between February 22, 2022 – April 21, 2022.
[b] This row of denominators only includes females who reported at least one long-term symptom.

© 2024 Shah MM et al. *JAMA Network Open.*

eTable 7: Physical and mental health in the last 4 weeks among American Red Cross blood donors surveyed during February 22, 2022 – April 21, 2022

	**History of SARS-CoV-2 infection, No (%) N = 83,015[1]**	**95% CI**	**No history of SARS-CoV-2 infection, No (%) N = 154,283[1]**	**95% CI**	**p-value[b]**
**In the past 4 weeks, would you say your general health was:**					<0.001
Excellent	20,448 / 81,108 (25.2%)	24.9-25.5	39,029 / 151,794 (25.7%)	25.5-25.9	
Very good	36,217 / 81,108 (44.7%)	44.3-45.0	72,845 / 151,794 (48.0%)	47.7-48.2	
Good	20,256 / 81,108 (25.0%)	24.7-25.3	35,229 / 151,794 (23.2%)	23.0-23.4	
Fair	3,764 / 81,108 (4.6%)	4.5-4.8	4,376 / 151,794 (2.9%)	2.8-3.0	
Poor	423 / 81,108 (0.5%)	0.5-0.6	315 / 151,794 (0.2%)	0.2-0.2	
Missing	1,907		2,489		
**In the past 4 weeks, did poor mental or physical health keep you from doing your usual activities, such as self-care, work, or recreation?**					<0.001
Yes, often	2,063 / 81,073 (2.5%)	2.4-2.7	2,320 / 151,769 (1.5%)	1.5-1.6	
Yes, sometimes	10,368 / 81,073 (12.8%)	12.6-13.0	14,905 / 151,769 (9.8%)	9.7-10.0	
No	67,733 / 81,073 (83.5%)	83.3-83.8	133,057 / 151,769 (87.7%)	87.5-87.8	
I don't know/am unsure	909 / 81,073 (1.1%)	1.1-1.2	1,487 / 151,769 (1.0%)	0.9-1.0	
Missing	1,942		2,514		
**How does your current physical health compare with your physical health prior to the pandemic?**					<0.001
Better than before the pandemic	6,608 / 81,096 (8.1%)	8.0-8.3	12,991 / 151,830 (8.6%)	8.4-8.7	
Worse than before the pandemic	13,413 / 81,096 (16.5%)	16.3-16.8	16,615 / 151,830 (10.9%)	10.8-11.1	
About the same	59,892 / 81,096 (73.9%)	73.5-74.2	120,899 / 151,830 (79.6%)	79.4-79.8	
I don't know/am unsure	1,183 / 81,096 (1.5%)	1.4-1.5	1,325 / 151,830 (0.9%)	0.8-0.9	
Missing	1,919		2,453		
**How does your current mental health compare with your mental health prior to the pandemic?**					<0.001
Better than before the pandemic	4,325 / 81,108 (5.3%)	5.2-5.5	7,740 / 151,810 (5.1%)	5.0-5.2	
Worse than before the pandemic	15,336 / 81,108 (18.9%)	18.6-19.2	22,781 / 151,810 (15.0%)	14.8-15.2	
About the same	60,021 / 81,108 (74.0%)	73.7-74.3	118,921 / 151,810 (78.3%)	78.1-78.5	
I don't know/am unsure	1,426 / 81,108 (1.8%)	1.7-1.9	2,368 / 151,810 (1.6%)	1.5-1.6	
Missing	1,907		2,473		

CI = confidence interval

[a] History of SARS-CoV-2 defined as reported confirmed infection since March 2020 (by polymerase chain reaction or antigen testing, serologic testing, or healthcare provider diagnosis) or anti-nucleocapsid antibody-positive during a blood donation since March 2020. Reported as n / N (%).

[b] Pearson's Chi-squared test

© 2024 Shah MM et al. *JAMA Network Open.*
