# Supplementary material for: Long-Term Symptoms Associated With SARS-CoV-2 Infection Among Blood Donors
Source: JAMA Netw Open. 2024 Apr 8;7(4):e245611. doi: 10.1001/jamanetworkopen.2024.5611 (PMC11002700; doi:10.1001/jamanetworkopen.2024.5611)
Supplement: Supplement 2. — Data Sharing Statement [file jamanetwopen-e245611-s002.pdf]

Data Sharing Statement

Shah. Long-Term Symptoms Associated With SARS-CoV-2 Infection Among Blood Donors.
JAMA Netw Open. Published April 08, 2024. doi:10.1001/jamanetworkopen.2024.5611

Data
Data available: No
